# Gingival phenotype assessment methods and classifications revisited: a preclinical study

**DOI:** 10.1007/s00784-021-03860-5

**Published:** 2021-03-16

**Authors:** Kai R. Fischer, Jasmin Büchel, Tiziano Testori, Giulio Rasperini, Thomas Attin, Patrick Schmidlin

**Affiliations:** 1 Clinic of Conservative and Preventive Dentistry, Division of Periodontology and Peri-implant Diseases, Centre of Dental Medicine, University of Zurich, Zurich, Switzerland; 2 Department of Periodontics and Oral Medicine, School of Dentistry, University of Michigan, Ann Arbor, MI USA; 3 IRCCS Orthopedic Institute Galeazzi, Milan, Italy; 4 Department of Biomedical, Surgical and Dental Sciences, University of Milan, Milan, Italy; 5 Clinic of Conservative and Preventive Dentistry, Centre of Dental Medicine, University of Zurich, Zurich, Switzerland

**Keywords:** Gingival phenotype, Probe transparency, Soft tissue thickness, Phenotype classification, Periodontal morphotype, Phenotype probe

## Abstract

**Objective:**

To compare gingival phenotype assessment methods based on soft tissue transparency on different backgrounds and assessor experience levels.

**Methods:**

For this purpose, 24 gingival specimens were retrieved from pig jaws with tissue thicknesses from 0.2 to 1.25 mm. Three methods were assessed: periodontal probe PCP12 (thin/thick), double-ended periodontal probe DBS12 (thin/moderate/thick) and colour-based phenotype probe CBP (thin/moderate/thick/very thick). Each sample was photographed with each probe underneath and categorized whether the probe was visible or not using different coloured backgrounds. To measure experience level influence, dentists, dental undergraduate students and laypersons (*n* = 10/group) performed the evaluation.

**Results:**

PCP12 probe showed a threshold between 0.4 and 0.5 mm. To distinct between thin and moderate thick gingiva, a comparable range for DBS12 was found while moderate thickness was between 0.5 and 0.8 mm and for thick above 0.8 mm. CBP also showed a comparable threshold of 0.5 mm for thin versus moderate as compared with the other methods; above 0.8 mm, predominantly a very thick tissue was measured. In general, the background colour had a minor impact on PCP12 and DBS12, and investigator experience showed no clear influence on GP assessment.

**Conclusion:**

Based on probe transparency and within the limitation of a preclinical study, we suggest GP differentiation into three entities: thin (< 0.5 mm; high risk), moderate (0.5–0.8 mm; medium risk) and thick (> 0.8 mm; low risk).

**Clinical relevance:**

All three GP assessment methods are easy to perform and seem to have a high predictive value with a three entities classification for DBS12 and CBP.

## Introduction

Gingival phenotypes (GP) are assumed to be associated with specific tissue characteristics and dental treatment outcomes. For example, teeth with a thin GP are at greater risk for developing gingival recessions [1] and a thin GP may react more delicately to surgery and heal less predictably when treating gingival recessions. Accordingly, as compared with thicker GPs, more pronounced ridge resorptions can be anticipated after tooth extractions [2]; hence, a thick GP is recommended as one key aspect to reduce the risk for mucosal recession after immediate implant placement [3]. Thick peri-implant tissues seem to be associated with significantly less bone loss [4] and thicker tissues are able to disguise different restorative materials [5].

Eghbali and co-workers [6] showed, however, regardless of clinical experience, the difficulty to visually distinguish between gingival phenotypes. Consequently, different methods were proposed based on the measurement of buccal gingival thickness to correctly assess gingival phenotypes. The simplest non-invasive method was described by Kan et al. [7] based on transparency of a periodontal probe through the gingival margin and has shown the highest predictability an being visible for a gingival thickness (GT) < 0.6 mm (thin GP) and being less or not visible for > 1.0 mm (thick GP). GTs in between were less distinguishable applying a dichotomous classification. Case selection is of utmost importance before immediate implant placement and immediate restoration. Recently, the 5-year outcome of immediate implants has been presented in the aesthetic zone after careful case selection with only thick GP based on the above-mentioned dichotomous classification [8]. Eight out of 17 patients presented with a severe aesthetic complication, e.g. > 1 mm mucosal recession, raising the question whether only thick GP were selected or also patients with an intermediate tissue thickness might have been included partially accounting for the high complication rate.

To overcome the shortcoming of a dichotomous classification, two novel methodologies with corresponding classifications have been developed and introduced. After artificially stratifying a group of young Caucasians into moderately and extremely thin/thick GP, highly statistical significant differences were seen for very thin vs. very thick groups; however, no more differences between the moderate groups were observed [9]. Following these observations, a novel double-ended periodontal probe was evaluated and allowed differentiation into thin (median GT: 0.43 mm), moderate and thick GP (median GT: 0.83 mm). Another GP assessment method based on the visibility of differently coloured probe tips has been presented recently [10]. This approach allowed differentiation between thin, moderate, thick and very thick GP and significant differences in soft tissue response have been observed after orthodontic treatment with more gingival recession and loss of keratinized tissue for thin GP.

To date, no comparative study evaluated different GP classification methods based on probe transparency in a reproducible and standardized fashion. Multiple insertions of different probes within the gingival sulcus might impair the objectivity and reproducibility of the GP assessment by causing swelling or bleeding. In vitro soft tissue transparency measurements have been evaluated already to determine minute colour changes caused by different dental materials assessing soft tissue samples in varying thickness harvested from pig jaws [5, 11]. This approach has been proven to be objective, reproducible and easy to standardize. As another advantage, tissue samples can be harvested in a constant fashion, and camera settings are independent of surrounding influences and photographs can be independently and blindly be assessed by different evaluators.

Therefore, this method has been adopted for the current investigation, in which we aimed to compare different gingival phenotype assessment methods based on soft tissue transparency. Our main objective was to compare three GP assessment methods and to compare their surrogate classifications in relation to direct soft tissue thickness measures. In addition, influence of different tooth shades and investigator experience levels has been evaluated. This study attempted to identify gingival thickness threshold levels corresponding with different GPs.

The hypothesis was that all three methods have specific gingival thickness thresholds and all methods can be applied regardless of assessor experience and/or background colour.

## Material and methods

### GP assessment tools and classifications

Three different GP assessment methods were used for this ex vivo experiment, each method having a different classification for the determination of GP.

A periodontal probe—exemplary within this study—PCP12 (Hu-Friedy, Chicago, IL, USA) allowing a dichotomous differentiation (thin/thick). Double-ended, colour—as well as tip-thickness-coded periodontal probe DBS12 (Deppeler SA, Rolle, Switzerland) enabling a classification into three categories (thin/moderate/thick). GP-specific tool CBP (Hu-Friedy) with a flat, coloured tip design divides gingival thickness into four subgroups (thin/moderate/thick/very thick).

### Sample preparation

Ten freshly slaughtered pork upper jaws were used to retrieve gingival tissue samples. The pigs must have been sacrificed no longer than 2 h before the in vitro tests started. These pigs were raised and slaughtered for food production per local standards for animal welfare. The study protocol did not in any way influence the premortal fate of the animals or the slaughtering process. Therefore, this investigation was not classified as an animal study, and the institutional ethics committee of University of Zurich did not have any objections to the protocol. To avoid possible structural changes in the tissue, the pig jaws were immediately stored in a cooling chamber until the start of the experiment. To avoid structural changes in the tissue, the removed samples were also kept moist in a 0.9% sodium-chloride solution and stored in a cooling chamber until used. With the help of a scalpel, a total of 24 pieces of tissue of different thicknesses were removed from the buccal gingiva of the pig’s jaw. Using a digital calliper (HOLEX, Munich, Germany), the removed tissue pieces were checked for their thickness with an accuracy of 0.01 mm. Sample thickness ranged from 0.2 to 1.25 mm. Sample thickness was measured three times: directly after harvesting, before and after taking pictures with the different probes underneath. All measurements have been in a maximum range of ± 0.02 mm. In order to imitate a clinical situation with different tooth surface colours, three different backgrounds in A2, A3 and A4 were made from polymethyl-methacrylate resin (PMMA; 20 × 20 × 2.5 mm; Enamel Plus Temp, Schütz Dental GmbH, Rosbach, Germany).

**Fig. 1:**
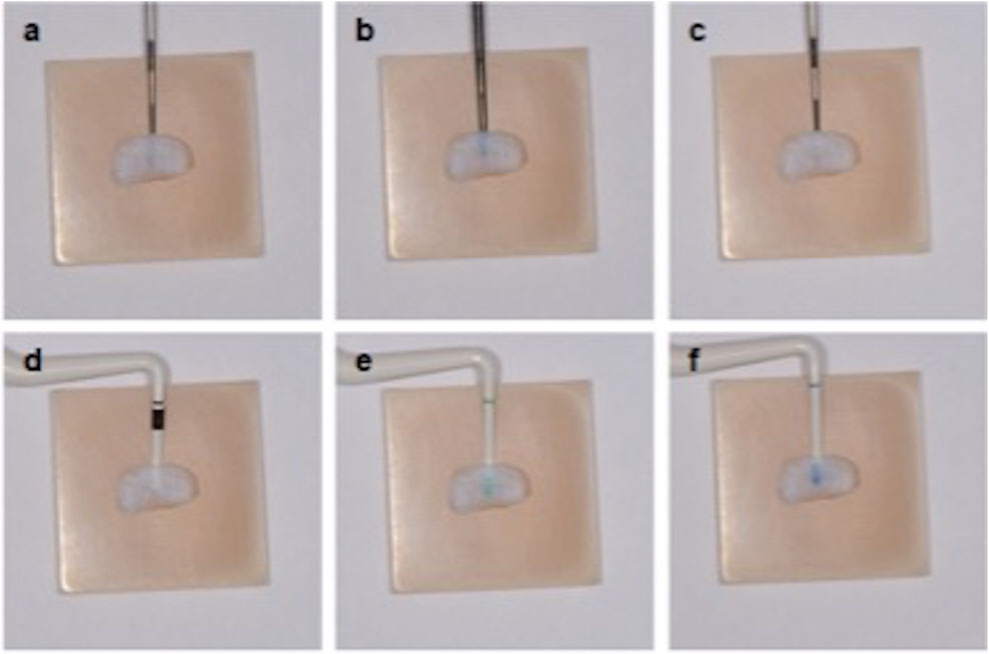
Examples of GP assessment (tissue sample of 0.22 mm thickness; background colour A4): **a** DBS 12 silver end, **b** DBS 12 black end, **c** PCP 12, **d** CBP white (#1), **e** CBP green (#2), **f** CBP blue (#3); note: all probes are visible through the tissue sample

To compare the three periodontal probes with each other, the gingival graft samples of different thicknesses were placed one after the other on the different backgrounds. Each probe tip was then inserted between the PMMA background as well as the gingival transplant to a depth of 3 mm and photographed (Fig. 1). With the help of a tripod, which allowed a precise distance between the camera and the gingival transplant of 300 mm comparable with working distance in daily dental practice, standardized pictures could be guaranteed. All pictures were taken with the D7100 SLR camera (Nikon, Minato, Tokyo, Japan) and the EM-140 DG macro flash unit (Sigma, Kawasaki, Kanagawa, Japan).

Four-hundred-thirty-two standardized photographs were taken and inserted in random order into a document from Microsoft PowerPoint 2019 (Microsoft, Redmond, Washington, USA). A total of thirty investigators (ten dentists, ten dental students and ten laypersons) categorized the photos independently of one another in terms of the visibility of the probe through the tissue (visible vs. non-visible).

### Statistical evaluation

All statistical analyses and plots were carried out with the statistical software R 4.0.3 including the packages tidyverse, ggplot2 and gplots. An inter-examiner agreement level of > 66.6% was set as a threshold level between two GP subgroups. To test the overall agreement between the different investigators independent of background colour, a uniform assessment threshold level was set to 90% of all investigators.

## Results

### GP threshold area

**Fig. 2:**
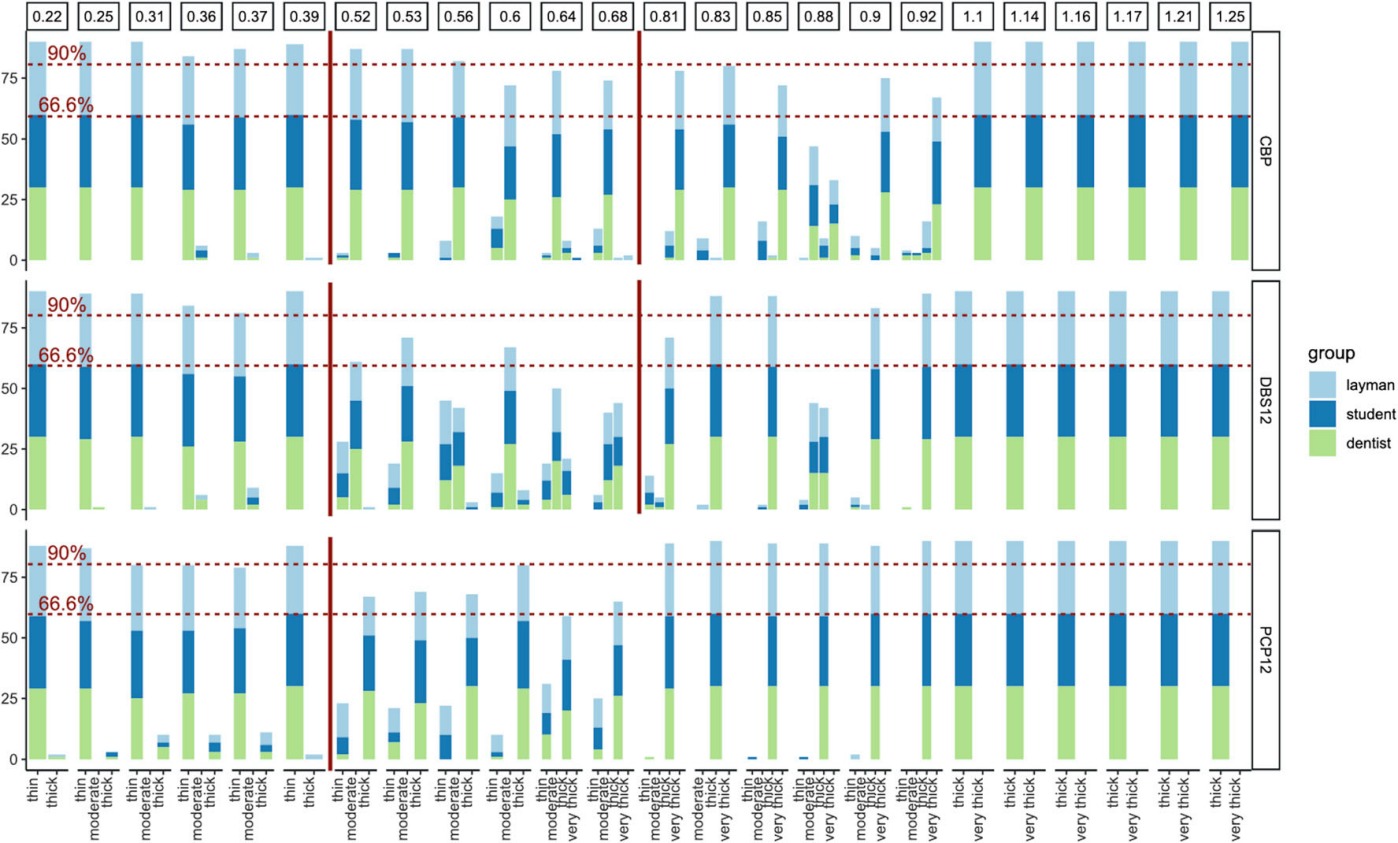
Bar graphs showing the results for GP assessment independent of background colour for CBP, DBS12 and PCP12; red lines indicating threshold levels between different GP (CBP: thin/moderate/very thick; DBS12: thin/moderate/thick; PCP12: thin/thick), threshold was set to 66.6% agreement between three investigator groups (lower dotted red line)

The results of all three backgrounds and all examiner groups were considered to assess a transition zone or threshold level for differentiation of gingival phenotype entities (Fig. 2).

### Influence of the soft tissue thickness

The soft tissue thickness range for determining the transition from a thin to a thick GP was found to range between 0.4 and 0.5 mm for PCP 12 periodontal probe.

With the help of the double-ended DBS 12, the gingival phenotype could be divided into three groups: thin, moderate and thick. The transition from a thin to a moderate phenotype could be observed with a thickness of the tissue samples also ranging between 0.4 and 0.5 mm, while a moderate to a thick phenotype changed with a thickness between 0.7 and 0.8 mm.

With the colour-coded CBP tips, the transition from a thin to a moderate phenotype was again seen in the range between 0.4 and 0.5 mm. A transition zone from “moderate” to “thick” could not be observed. Instead, a very thick phenotype was seen starting from 0.7 to 0.8 mm.

### Influence of the background colour

**Fig. 3:**
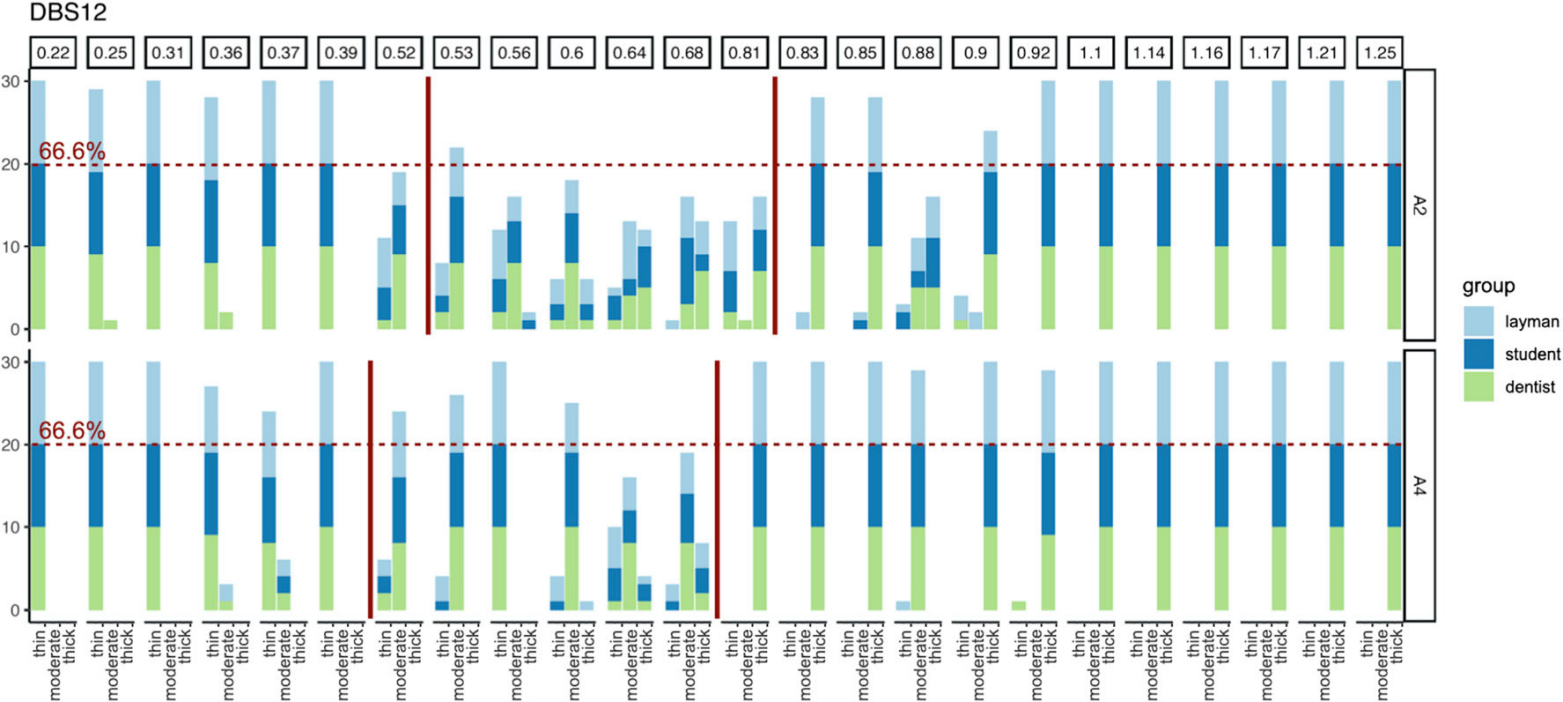
Bar graph showing the GP evaluation for DBS12 comparing two different background colours A2 versus A4; the darker background seems to improve differentiation to moderate GP, while shifting the 66.6% threshold to slightly lower values (A2: > 0.52/> 0.81 versus A4: > 0.39/> 0.68)

Evaluating the results for CPB, no tendency or clear pattern was observed between the three background options. To some extent, however, a darker background seemed to shift the decision between thin vs. moderate as well as moderate vs. thick for DBS12 and the moderate GP area was less indifferent possibly due to the higher contrast (Fig. 3). A similar observation was made for PCP12 and differentiation of thin vs. thick GP (A2: 0.5–0.55 mm versus A4: 0.4–0.5 mm).

### Influence of the investigator experience

No pattern could be observed, which identified differences based on investigator experience. Hence, further detailed presentation of this data was discarded.

### Investigator agreement

For PCP12, an agreement level of ≥ 90% between all participants was seen for soft tissue thicknesses ≤ 0.25 mm (thin GP) and ≥ 0.8 mm (thick GP). For CPB, the 90% level was reached for a thin GP ≤ 0.4 mm, for moderate GP 0.5–0.55 mm and very thick ≥ 1.1 mm; however, no such interval was seen for thick GP. For DBS12, ≥ 90% of all participants choose the same classification for thin GP ≤ 0.4 mm, for thick GP ≥ 0.8 mm and, as partially seen in Fig. 2, agreement for moderate GP is less univocal between 0.5 and 0.8 mm.

## Discussion

This study compared three gingival phenotype assessment methods based on transparency through the gingival sulcus and, if possible, aimed to propose a clinically relevant and reproducible classification with corresponding clinical gingival thickness. Furthermore, different investigator groups as well as tooth colour have been chosen as variables. Applying a double-ended periodontal probe (DBS12) or a colour-coded assessment tool (CBP), it seems possible to differentiate between thin (≤ 0.5 mm), moderate and thick (≥ 0.8 mm) gingival phenotypes independent of tooth shade or experience level.

The 2017 World Workshop on the Classification of Periodontal and Peri-Implant Diseases and Conditions did recommend the use of a traditional periodontal probe based on probe visibility shining through the gingival tissue to differentiate between a thin (≤ 1 mm tissue thickness) and a thick GP (≥ 1 mm) [12]. Differently designed periodontal probes are commercially available with different colour schemes and tip thickness; consequently, different results might be seen clinically and a standardized method might be preferable. Earlier data obtained from a Caucasian population [9, 13, 14] together with this ex vivo report, however, are in contrast with the proposed threshold and do question the application of a simple dichotomous approach. In 2014, GP was evaluated before direct tissue thickness measurement with a pressure-controlled digital calliper—for the first time to the authors knowledge and later used by Liu, Pelekos and Jin [15]—and GP border was around 0.5 mm in young Caucasians [9]. Liu, Pelekos and Jin [15] did not apply a transparency method and set an artificial threshold at 1 mm in a Chinese population. Later, a modified double-ended periodontal probe to allow differentiation into thin/moderate/thick GP [13]. Again, GP was first assessed applying the transparency approach and GT was measured afterwards. Median GT for thin GP was 0.43 mm and for thick GP 0.83 mm supporting our current findings and proposed classification. Another study, however, failed to identify a gingival thickness threshold that could discriminate between thin versus thick GP based on a dichotomous method; nevertheless, a comparable difference in buccal bone and buccal soft tissue thickness was observed [16]. Gingival thickness therefore seems to be directly correlated to the underlying bone thickness and alveolar crest position; hence, pre-surgical evaluation is of high importance before, e.g. immediate implant placement [17].

The International Team of Implantology (ITI) SAC Assessment Tool already differentiates between three GPs to identify high-, medium- and low-risk patients before implant placement and subsequent prosthetic treatment [18]. Trans-gingival probing with either a periodontal probe or an endodontic instrument, ultrasonography or cone beam computer tomography (CBCT) are possible alternatives to the described probe transparency methods; however, these other techniques are either invasive, not available to most clinicians or rather technique sensitive. Probe transparency, in contrast, was recommended as an easy-to-perform, low-cost and non-invasive method for daily clinical practice [19]. Using a simple dichotomous classification as seen for PCP12 (< 0.5 mm >), only high-risk patients with a thin GP could be evaluated. If a more comprehensive approach is warranted, DBS12 or CBP might allow a more advanced differentiation into three subgroups with similar prediction for tissue thickness. Furthermore, distinction between thick and very thick for CBP might not be possible or clinically relevant because of the congruent thickness appraisals for the blue and green tool tip. Supporting this assumption, similar clinical results have been shown for thick and very thick GP based on CBP classification after root coverage procedures without grafts showing; however, no statistically significant differences [20]. Coronally advanced flap procedures yielded complete root coverage in 20% of thin GP, 60% of moderate and ≥ 80% for thick/very thick GP [21]. Future research needs to evaluate treatment protocols based on GP before, e.g. root coverage procedures, implant or orthodontic treatment.

The present study did not evaluate differences of gingival colour, which may be considered as a shortcoming. Potentially, only slight variations within tissue samples could impair the ability to evaluate probe visibility. Tissue samples were strictly kept moist and cooled during the whole investigation; however, slight changes in tissue thickness or colour still might have been occurred. In addition, multiple intraoral measurements at the same location—e.g. six times for our study—might lead to tissue changes as well. While we looked for a high investigator agreement threshold level (≥ 90%) instead of inter-rater reliability or correlation due to inherent statistical calculation and interpretation shortfalls, we did not check intra-rater reliability as another study limitation. In addition, the study assessed non-vital tissues without blood flow which might account for differences to clinical measurements. It remains unclear to which extend gingival pigmentation, collagen content, blood flow and circulation characteristics or surface texture might influence GP assessment. GP differences among ethnicities still need to be evaluated. Furthermore, the influence of different diameter of instrument tips causing different pressure on the gingival margin or within the sulcus cannot be assessed ex vivo. Clinical studies need to confirm the findings of this preclinical investigation.

## Conclusion

PCP12, DBS12 and CBP seem to be easy-to-use and reliable tools for daily clinical practice. Based on the above presented preclinical and previous clinical findings of our group, differentiation in more than thin vs. thick GP seems feasible and is advocated. GP classification might be thin (< 0.5 mm, high risk), moderate (0.5–0.8 mm, medium risk) and thick (> 0.8 mm, low risk). Further clinical investigation and clinical trials are needed to approve the above mentioned thresholds and to evaluate the impact on different treatment protocols.
